# How do apathetic and depressive symptoms relate to functional capacity? A cross-sectional survey among community-dwelling middle-aged and older adults in Japan

**DOI:** 10.1186/s12889-024-19091-8

**Published:** 2024-11-14

**Authors:** Hikaru Oba, Akira Kanda, Kazushige Ihara, Asano Tanabu, Hiroshi Shimoda

**Affiliations:** 1 Graduate School of Health Sciences, Hirosaki University, Aomori, Japan; 2 Department of Nutrition, Faculty of Health Sciences, Graduate School of Health Sciences, Aomori University of Health and Welfare, Aomori, Japan; 3 Department of Social Medicine, Graduate School of Medicine, Hirosaki University, 5, Zaifu-cho, Aomori, 036-8562 Japan; 4 Department of Anatomical Science and Neuroanatomy, Cell Biology and Histology, Graduate School of Medicine, Hirosaki University, Aomori, Japan

**Keywords:** Apathy, Depression, Older adults, Interaction, Functional capacity

## Abstract

**Background:**

Apathetic and depressive symptoms are frequently observed among older adults, and are risk factors for functional decline and dementia progression. However, how these symptoms influence functional capacity remains unclear. This study investigated the relationship between apathetic and depressive symptoms and functional capacity, considering the interaction between apathetic and depressive symptoms.

**Methods:**

A cross-sectional questionnaire survey targeting community dwelling middle-aged or older adults was conducted. We sent a questionnaire to 984 individuals and received 320 responses. Data with missing values and participants aged under 50 were excluded, resulting in 212 participants (88 men and 124 women, mean age = 73.4 ± 9.3 years). Apathetic symptoms were evaluated using the Dimensional Apathy Scale (J-DAS), which comprises executive, emotional, and cognitive and behavioral initiation aspects of apathy. Depressive symptoms were evaluated using the Geriatric Depression Scale (GDS). Functional capacity was assessed using the Japan Science and Technology Agency Index of Competence, which comprises technology usage, information practice, life management, and social engagement.

**Results:**

Mean score of each J-DAS factor and GDS was 5.3 ± 3.4 (executive), 12.0 ± 3.0 (emotional), 11.8 ± 5.1 (initiation), and 4.5 ± 3.3, respectively. The emotional and initiation aspects of J-DAS were significantly associated with information practice (β = -0.15, *p* < .05 for emotional; β = -0.27, *p* < .001 for initiation) and life management (β = -0.20, *p* < .01 for emotional; β = -0.22, *p* < .01 for initiation) in functional capacity. GDS was associated only with social engagement (β = -0.31, *p* < .001). Although the interaction between the initiation factor of J-DAS and GDS was significantly associated with life management (β = -0.16, *p* < .05), the *R*^2^ change was insignificant. The emotional factor of J-DAS was associated with technology usage (β = -0.13, *p* < .05), although less strongly than age. The executive factor of J-DAS had insignificant associations with all aspects of functional capacity.

**Conclusions:**

Apathetic and depressive symptoms are independently, rather than interactively, associated with different aspects of functional capacity. As older adults with apathetic or depressive symptoms might struggle to seek help from public health services, they should be targeted with active interventions from healthcare professionals.

## Background

Globally, the number of older adults has been increasing with the improvement of nutrition and healthcare. Successful aging, characterized by physical, mental, and social health [1], is a key goal not only for individuals but also for national policy, given that increasing medical and care costs for older adults has become a serious issue worldwide [2]. However, data from the World Health Organization Global Health Observatory showed that the global average for healthy life expectancy (63.7 years) was approximately 10 years shorter than total life expectancy (73.3 years) in 2019 [3]. To bridge this gap and achieve successful aging, prevention of physical, psychological, and functional decline is crucial.

Traditionally, functional capacity required to maintain daily life has been divided into two aspects [4]. First, basic activities of daily living (ADL) comprise the ability to achieve physical self-maintenance (such as feeding, bathing, and dressing). Second, instrumental ADL includes more complex activities, including shopping, cooking, and food preparation. Lawton & Brody [4] developed the Physical Self-Maintenance Scale and Instrumental Activities of Daily Living Scale to assess both forms of ADL, which are used globally. In Japan, the Tokyo Metropolitan Institute of Gerontology Index of Competence (TMIG-IC) [5] has also been employed to assess the higher-level capacity of older adults. It is based on the stages of instrumental self-maintenance, effectance, and social role from Lawton’s seven-stage hierarchical model of competence [6].

Recent Japanese research has developed a new scale, the Japan Science and Technology Agency Index of Competence (JST-IC) [7, 8], that aims to identify the functional capacities older adults need to adapt to the current environment, which has changed through the decades since Lawton’s scale and the TMIG-IC were developed. The JST-IC extended the concepts of functional capacity in Lawton’s scale and the TMIG-IC to include higher-level and more complex skills (e.g., using electronic devices and preventing oneself from becoming a victim of crime). Decline in higher-level capacities is observed earlier than in basic ADL; such a decline may be an early sign of dementia [9]. Decline in higher-level competence was also associated with cognitive frailty, defined as comorbid physical frailty and cognitive impairment [10].

Apathy is among the health-threatening factors in later life. Apathy is defined as loss of motivation [11], which is divided into three domains including goal-directed behavior, goal-directed cognitive activity, and emotion [12]. Other criteria focus on the objective measurement of apathy, which define apathy as diminished self-generated voluntary and purposeful behavior [13]. Goal-directed behavior encompasses three aspects including cognition, emotion, and initiation [13]. Updated criteria proposed in 2018 [14] also focused on goal-directed behavior rather than motivation, which defined apathy as “a quantitative reduction of goal-directed activity either in behavioral, cognitive, emotional or social dimensions in comparison to the patient’s previous level of functioning in these areas.” The prevalence rate of apathetic symptoms among community-dwelling adults aged 50 years and above was 23.7% in the US; apathy was associated with the decline of instrumental functions [15].

Depression is another key factor associated with late-life health as well as apathy. Depression is characterized by a depressive mood and loss of interest or pleasure [16]. The prevalence of major depressive disorder using Composite International Diagnostic Interview among community-dwelling middle aged-older adults was 4.0% in the US; this rate declined with age [17]. A previous longitudinal survey showed the effect of the interaction between cortical amyloid and depressive symptoms on cognitive function [18]. A recent systematic review and meta-analysis indicated the relationship between depressive symptoms and frailty [19].

The above studies suggest that both apathetic and depressive symptoms relate to serious physical and psychological decline [15, 18, 19]. However, although these symptoms often coexist, they are considered distinct conditions [13]. For instance, apathetic symptoms without depressive symptoms were associated with progression to dementia; this was not the case for apathy in the context of depressive affect or depressive symptoms without apathy [20]. Further, older adults with both apathetic and depressive symptoms, rather than those with depressive symptoms only, have an increased risk of transitioning from mild cognitive impairment to Alzheimer’s disease [21].

In summary, apathetic and depressive symptoms clearly influence functional capacity. Previous studies suggest that these have different effects [20, 21]; the conceptual difference between these conditions may lead to heterogeneous effects on functional capacity. However, the effect of the interaction of these symptoms remains unclear. This study investigates whether apathetic and depressive symptoms are independently or interactively related to functional capacity.

## Methods

### Design and setting

We conducted a survey on apathetic symptoms, depressive symptoms, and functional capacity among community dwelling middle-aged and older adults from November 2020 to March 2021. We recruited people who belonged to a medical volunteer organization in Aomori prefecture, Japan. Aomori prefecture is a northern area in Japan, where the proportion of the population aged 65 years was 33.3% in 2020 [22]. In total, 984 individuals received the questionnaire by mail and 320 responded (response rate = 32.5%). The Ethics Committee of Hirosaki University School of Medicine approved the study (number: 2020 − 109).

### Measures

#### Demographic characteristics

We collected demographic data relating to age, sex, education level (1. elementary school, 2. junior high-school, 3. high-school, or 4. vocational school, two-year college, or university), current working status (0. not working, (1) working), and subjective health (1. very good, (2) good, (3) not good, (4) bad).

#### Apathy and depression

To assess apathetic symptoms, we employed the Japanese version of the Dimensional Apathy Scale (J-DAS) [23, 24]. This 24-item scale comprises three factors including executive, emotional, and cognitive and behavioral initiation. These factors are based on Levy and Dubois’ definition of apathy [13]. The executive factor (e.g., I need a bit of encouragement to get things started) is involved in planning and acting, such as organizing goals. The emotion factor (e.g., I express my emotions) relates to the emotional aspects associated with behavior. The initiation factor (e.g., I contact my friends) relates to self-activated thoughts or behaviors. Each factor includes eight items scored on 4-point rating scales ranging from 0 (hardly ever) to 3 (almost always). Scores for each factor range from 0 to 24, with higher scores indicating greater apathetic symptoms.

Depressive symptoms were evaluated using the 15-item version of the Geriatric Depression Scale (GDS) [25, 26]. GDS is a self-rated scale based scored using dichotomous response options (Yes or No). The score range is 0–15, with higher scores indicating greater depressive symptoms, and scores over 10 suggesting the presence of depression [25].

### Functional capacity

Functional capacity in daily life was evaluated using the JST-IC [7, 8]. This scale has 16 items across four factors including technology usage (e.g., Can you use a mobile phone?), information practice (e.g., Are you interested in news and events from overseas?), life management (e.g., Do you follow any measures to prevent yourself from becoming a victim of crime?), and social engagement (e.g., Do you participate in regional festivals or events?). Each factor includes four items and is answered using dichotomous variables (Yes or No). Scores for each factor range from 0 to 4, with higher scores indicating greater functional capacity.

### Statistical analysis

First, descriptive characteristics were calculated. Subsequently, one-way analysis of variance (ANOVA) was performed to assess intrapersonal differences in the factors of JST-IC and J-DAS. Univariate correlations between J-DAS, GDS, and JST-IC were calculated using Pearson’s correlation analysis. P-values were adjusted using Holm’s method. Lastly, hierarchical multiple regression analysis was performed to reveal the factors associated with functional capacity. In Step 1, we entered the demographic characteristics, J-DAS factors, and GDS scores independently into the regression. Subsequently, the interaction variable of each J-DAS factor and GDS was added. The interaction variables were centered to prevent multicollinearity. All statistical analyses were performed using R version 4.0.3 [27], and Pequod package [28].

## Results

**Fig. 1:**
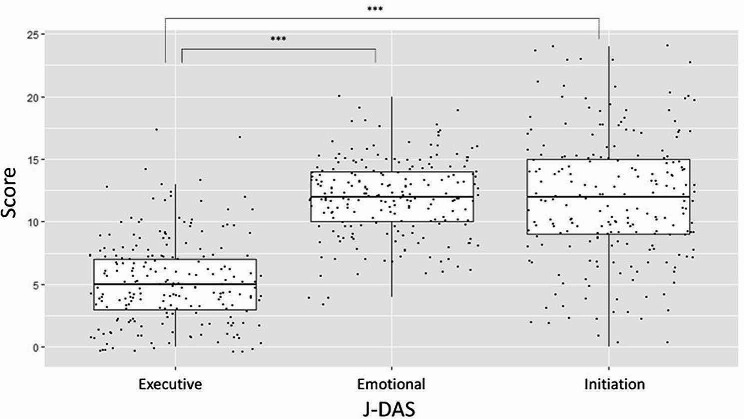
Comparison of J-DAS sub-factors. *Note* J-DAS = Dimensional Apathy Scale (Japanese version). Dots indicate individual scores. ^***^p < .001

**Fig. 2:**
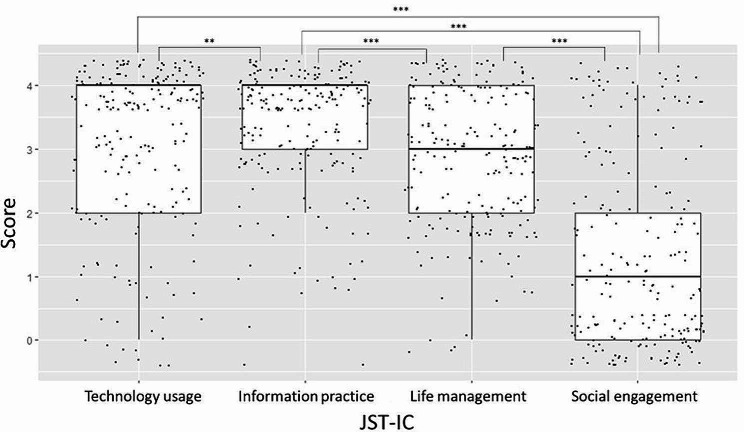
Comparison of JST-IC sub-factors. *Note* JST-IC = Japan Science and Technology Agency Index of Competence. Dots indicate individual scores. ^**^p < .01 ^***^p < .001

Data that included missing values or related to people aged under 50 years were excluded; as a result, data from 212 participants (88 men and 124 women, mean age = 73.4 years, SD = 9.3 years) were analyzed. Table 1 shows the participants’ descriptive characteristics. Figures 1 and 2 show the boxplot distributions of J-DAS and JST-IC scores, respectively, arranged by their sub-factors. The one-way ANOVA showed a significant difference in mean scores among the J-DAS factors, F (2, 635) = 237.5, *p* < .001. Post hoc analysis also revealed that the mean score of the executive factor of J-DAS was lower than that of the other two factors (executive: M = 5.3, SD = 3.4; emotion: M = 12.0, SD = 3.0; initiation: M = 11.8, SD = 5.1; ps < 0.001). The mean scores also significantly differed among the sub-factors of JST-IC, F (3, 847) = 142.3, *p* < .001. Post hoc analysis showed that the mean scores for the social engagement factor were the lowest while scores for information practice were the highest (information practice: M = 3.3, SD = 1.0; technology usage: M = 3.1, SD = 1.3; life management: M = 2.9, SD = 1.0; social engagement: M = 1.3, SD = 1.5). There was no difference in the mean scores between the technology usage and life management factors.

**Table 1:** Participants’ descriptive characteristics

	M	SD	Min-Max
Age	73.4	9.3	52–96
JST_IC			
Technology usage	3.1	1.3	0–4
Information practice	3.3	1.0	0–4
Life management	2.9	1.0	0–4
Social engagement	1.3	1.5	0–4
J-DAS			
Executive	5.3	3.4	0–17
Emotional	12.0	3.0	3–20
Initiation	11.8	5.1	0–24
GDS	4.5	3.3	0–14
	***N***	***%***	
Sex			
Men	88	41.5	
Women	124	58.5	
Education level			
Junior high school	27	12.7	
High school	103	48.6	
University	82	38.7	
Current work status			
Working	68	32.1	
Not working	144	67.9	

*Note* JST-IC = Japan Science and Technology Agency of Index for Competence; J-DAS = Dimensional Apathy Scale (Japanese version); GDS = Geriatric Depression Scale.

Univariate correlation analysis between J-DAS, GDS, and JST-IC revealed relationships among the scales (Table 2). The executive factor of J-DAS was related to the life management (*r* = -.22, *p* < .01) and social engagement (*r* = -.20, *p* < .01) factors of JST-IC. The emotional factor of J-DAS was related to the technology usage (*r* = -.19, *p* < .01), information practice (*r* = -.17, *p* < .05), and life management (*r* = -.24, *p* < .001) factors of JST-IC. The initiation factor of J-DAS was related to all JST-IC factors (technology usage: *r* = -.16, *p* < .05; information practice: *r* = -.31, *p* < .001; life management: *r* = -.36, *p* < .001; social engagement: *r* = -.26, *p* < .001). The GDS was related to the information practice (*r* = -.14, *p* < .05), life management (*r* = -.28, *p* < .001), and social engagement (*r* = -.36, *p* < .001) factors of JST-IC.

**Table 2:** Pearson’s correlation between JST-IC, J-DAS, and GDS

	JST-IC			
	Technology usage	Information practice	Life management	Social engagement
J-DAS				
Executive	-0.13	-0.09	-0.22^**^	-0.20^**^
Emotional	-0.19^**^	-0.17^*^	-0.24^***^	-0.04
Initiation	-0.16^*^	-0.31^***^	-0.36^***^	-0.26^***^
GDS	-0.11	-0.14^*^	-0.28^***^	-0.36^***^

*Note p*-values are adjusted by Holm’s method; JST-IC = Japan Science and Technology Agency of Index for Competence; DAS = Dimensional Apathy Scale (Japanese version); GDS = Geriatric Depression Scale. ^*^p < .05 ^**^p < .01 ^***^p < .001

Table 3 shows the factors associated with functional capacity. Hierarchical multiple regression analysis adjusted for demographic characteristics showed that the J-DAS emotion factor was significantly associated with the technology usage, information practice, and life management factors of JST-IC (technology usage: β = -0.13, *p* < .05; information practice: β = -0.15, *p* < .05; life management: β = -0.20, *p* < .01). The J-DAS initiation factor was significantly associated with the information practice and life management factors of JST-IC (information practice: β = -0.27, *p* < .001; life management: β = -0.22, *p* < .01). Moreover, as the interaction between the J-DAS initiation factor and GDS was significant, we conducted simple slope analysis. The result showed that the J-DAS initiation factor was significantly associated with the life management factor of JST-IC when the GDS score was more than 1 SD above the mean (b = -0.09, *p* = .002). However, delta *R*^*2*^ (indicating the increasing ratio of variance from step 1) was insignificant. The GDS was significantly associated with the social engagement factor of JST-IC (β = -0.31, *p* < .001).

**Table 3:** Factors associated with functional capacity

JST-IC	Technology usage		Information practice	
	STEP 1	STEP 2	STEP 1	STEP 2
Age	**-0.52 [-0.66, -0.38]** ^***^	**-0.51 [-0.66, -0.37]** ^***^	0.16 [0, 0.31]	**0.17 [0.01, 0.33]**
Sex	-0.02 [-0.14, 0.10]	-0.03 [-0.15, 0.10]	-0.06 [-0.19, 0.07]	-0.06 [-0.20, 0.08]
Subjective health	-0.07 [-0.21, 0.07]	-0.07 [-0.20, 0.07]	0.03 [-0.12, 0.19]	0.05 [-0.11, 0.20]
Work status	0.04 [-0.10, 0.18]	0.04 [-0.10, 0.18]	0.03 [-0.12, 0.18]	0.03 [-0.13, 0.18]
High school [ref. under junior high school]	0.02 [-0.17, 0.21]	0.03 [-0.16, 0.22]	0.14 [-0.08, 0.35]	0.13 [-0.09, 0.34]
University	0.01 [-0.19, 0.20]	0.02 [-0.17, 0.22]	0.16 [-0.05, 0.38]	0.16 [-0.05, 0.38]
J-DAS executive	0.00 [-0.14, 0.15]	-0.03 [-0.19, 0.12]	0.00 [-0.16, 0.16]	-0.01 [-0.18, 0.16]
J-DAS emotional	-0.13 [-0.25, 0]	**-0.13 [-0.25, -0.01]** ^*^	**-0.14 [-0.28, -0.01]** ^*^	**-0.15 [-0.29, -0.01]** ^*^
J-DAS initiation	-0.06 [-0.20, 0.08]	-0.05 [-0.19, 0.10]	**-0.28 [-0.44, -0.13]** ^***^	**-0.27 [-0.43, -0.11]** ^***^
GDS	-0.12 [-0.27, 0.04]	-0.13 [-0.29, 0.03]	-0.02 [-0.19, 0.16]	-0.03 [-0.21, 0.15]
J-DAS executive * GDS		0.09 [-0.03, 0.20]		0.02 [-0.11, 0.15]
J-DAS emotional * GDS		0.02 [-0.10, 0.13]		-0.02 [-0.14, 0.11]
J-DAS initiation * GDS		-0.04 [-0.16, 0.08]		0.04 [-0.09, 0.17]
*F* (*df*)	9.15 (10, 201)^***^	7.19 (13, 198)^***^	3.47 (10, 201)^***^	2.68 (13, 198)^**^
*R* ^*2*^	0.31	0.32	0.15	0.15
Adj *R*^*2*^	0.28	0.28	0.10	0.09
*ΔR* ^*2*^		0.01		0.00
**JST-IC**	**Life management**		**Social engagement**	
	**STEP 1**	**STEP 2**	**STEP 1**	**STEP 2**
Age	-0.04 [-0.19, 0.11]	-0.06 [-0.21, 0.09]	-0.01 [-0.17, 0.15]	0.01 [-0.15, 0.17]
Sex	0.09 [-0.04, 0.21]	0.06 [-0.07, 0.19]	-0.09 [-0.22, 0.05]	-0.08 [-0.22, 0.05]
Subjective health	-0.07 [-0.21, 0.08]	-0.09 [-0.23, 0.06]	0.00 [-0.15, 0.15]	0.01 [-0.14, 0.16]
Work status	0.07 [-0.08, 0.22]	0.09 [-0.06, 0.23]	-0.01 [-0.17, 0.14]	-0.02 [-0.17, 0.14]
High school	-0.08 [-0.28, 0.12]	-0.04 [-0.24, 0.17]	0.09 [-0.12, 0.3]	0.10 [-0.12, 0.32]
University	0.00 [-0.20, 0.20]	0.03 [-0.18, 0.23]	0.05 [-0.16, 0.26]	0.07 [-0.14, 0.29]
J-DAS executive	-0.07 [-0.22, 0.08]	-0.10 [-0.26, 0.07]	0.01 [-0.15, 0.17]	-0.03 [-0.20, 0.14]
J-DAS emotional	**-0.21 [-0.34, -0.08]** ^**^	**-0.20 [-0.33, -0.07]** ^**^	-0.05 [-0.18, 0.09]	-0.06 [-0.20, 0.08]
J-DAS initiation	**-0.21 [-0.36, -0.06]** ^**^	**-0.22 [-0.37, -0.06]** ^**^	-0.14 [-0.3, 0.01]	-0.12 [-0.28, 0.04]
GDS	-0.16 [-0.33, 0]	-0.14 [-0.31, 0.03]	**-0.30 [-0.47, -0.12]** ^***^	**-0.31 [-0.49, -0.14]** ^***^
J-DAS executive * GDS		0.06 [-0.06, 0.19]		0.09 [-0.04, 0.22]
J-DAS emotional * GDS		0.00 [-0.12, 0.13]		0.07 [-0.06, 0.20]
J-DAS initiation * GDS		**-0.16 [-0.28, -0.03]** ^*^		-0.02 [-0.15, 0.12]
*F* (*df*)	5.54 (10, 201)^***^	4.85 (13, 198)^***^	3.66 (10, 201)^***^	3.03 (13, 198)^***^
*R* ^*2*^	0.22	0.24	0.15	0.17
Adj *R*^*2*^	0.18	0.19	0.11	0.11
*ΔR* ^*2*^		0.02		0.02

*Note* J-DAS = Dimensional Apathy Scale (Japanese version); GDS = Geriatric Depression Scale; JST-IC = Japan Science and Technology Agency Index of Competence. Bold type highlights significant effects. ^*^p < .05 ^**^p < .01 ^***^p < .001

## Discussion

The current findings demonstrate that apathetic and depressive symptoms were independently associated with functional capacity and that the strength of these associations varied among the different factors. Apathetic symptoms were associated with all functional capacity factors except for social engagement. Therefore, depressive symptoms were the only factor associated with social engagement. The results also suggested that although the effect size was small, both symptoms had an interactive effect on social engagement. These findings suggest that apathetic and depressive symptoms are independently associated with functional decline rather than having an interactive impact. Therefore, intervening in depressive symptoms as well as the emotional and initiation aspects of apathetic symptoms may contribute to preventing the decline of functional capacity.

The emotional and initiation aspects of apathetic symptoms were associated with the information practice and life management factors of functional capacity. Emotions are important for motivating behavior. For instance, positive emotions such as empathy motivate prosocial behavior [29, 30]. The information practice factor comprises taking an interest in international news, watching movies or listening to music, and making decisions about the credibility of health-related information, while the life management factor comprises providing care to family members. These behaviors are required to activate emotion and voluntary thought, including taking interest in things and showing empathy toward others, which likely explains their role in the findings. Moreover, although the *R*^*2*^ change was not, the interaction coefficient between apathetic and depressive symptoms was significantly related to life management. This result is attributable to a lack of statistical power. Therefore, further surveys are necessary to reveal the effect of apathetic and depressive symptoms on life management. Nonetheless, this finding suggests that higher apathetic and depressive symptoms may cause a more severe decrease in life management.

The emotional aspect of apathetic symptoms was also associated with technology usage. A previous longitudinal study suggested that internet use predicts cognitive function after two years [31]. Internet use is also beneficial in terms of building communication and social connectedness, which are considered to improve older adults’ quality of life [32]. Our findings suggest that apathetic symptoms result in a decline in the frequency of internet use through digital devices such as mobile phones. However, the effect of apathetic symptoms on technology usage was smaller than that of age. The Ministry of Internal Affairs and Communications [33] in Japan has shown that although the utilization rate of older adults has been increasing in the past five years, the rate of internet usage decreases with age. Notably, the items relating to technology usage in the JST-IC comprise whether the respondent is able to use a mobile phone or send an e-mail; in other words, these items evaluate older adults’ capacity to use these devices rather than actual usage frequency. As these traits (i.e., capacity to use technology) do not change in the short term, the participants’ cohort effect could be larger than the impact of apathetic symptoms.

As mentioned above, social engagement was associated only with depressive symptoms, rather than apathetic symptoms, showing a different tendency when compared to other factors of JST-IC. The particularity of the social engagement factor is that it refers to social rather than solitary activities. Social engagement is required to construct active relationships with neighbors and the community. One of the differences between apathetic and depressive symptoms is negative emotionality [34, 35], where depression leads to believing that one cannot do things or feeling guilty for being an inconvenience or burden to others. People with depression are more likely to actively avoid social activity than those with apathy [34]. People with depressive symptoms without apathetic symptoms also exhibit higher levels of depressive mood and fatigue than those with apathetic symptoms [36]. Therefore, emotional distress among patients with depressive symptoms might decrease social engagement.

The executive factor of the J-DAS was not associated with any functional capacity factors. The executive aspect of apathetic symptoms involves the elaboration of action plans, such as set-shifting or rule settings. One of the characteristics observed among people with executive apathetic symptoms is slow response after stimulation [13]. The JST-IC, which evaluates the status of daily activities, does not necessarily reflect detailed aspects of cognitive processing. Moreover, the score of the executive factor was lower than those of the other factors. Given the low scores for this factor, it appears this aspect of apathetic symptoms was less prominent among participants, explaining the lack of association with the JST-IC factors.

This study has several limitations. First, the survey design was cross-sectional; therefore, the causal relationship among apathetic symptoms, depressive symptoms, and functional capacity remains unclear. A longitudinal survey is necessary to clarify our findings. Second, although participants’ ages ranged from 52 to 96 years, we did not investigate their actual physical and cognitive function. People with frailty and mild cognitive impairment or dementia that is associated with decline in functional capacity [9, 10] might have been included as participants. This limitation was caused by our employing a mail survey, making it difficult to conduct medical consultations or objective tests to evaluate physical and cognitive function. Third, this study identifies the symptoms of apathy and depression in a community, rather than clinical setting. The mean score of GDS was below the cutoff value and J-DAS scores were not too high compared with the other community samples in Japan [24, 25]. Therefore, most participants in this study may not suffer from clinically significant depression or apathy. The association between functional capacity and the symptoms in this study should be examined in clinical institutions to generalize our findings to patients with depression or apathy. Forth, we should cautiously interpret the results considering selection bias since the survey was conducted on a group of people in a specific region. Overall, further research with an improved survey design is necessary to reveal the relationship among apathy, depression, and functional capacity in more detail.

## Conclusions

Our findings showed that apathetic and depressive symptoms are independently associated with different functional capacities. Owing to emotional issues, older adults with apathy or depression might find it difficult to utilize public health services because they struggle to behave proactively. The lack of support resulting from apathy or depression may worsen older adults’ health problems. Therefore, active interventions by professionals such as public health nurses or social workers should be conducted among older adults experiencing these symptoms.

## Data Availability

The datasets used and/or analyzed during the current study are available from the corresponding author on reasonable request.

## Abbreviations

ADL: Activities of Daily Living
ANOVA: Analysis of Variance
GDS: Geriatric Depression Scale
J-DAS: Dimensional Apathy Scale (Japanese version)
JST-IC: Japan Science and Technology Agency of Index
TMIG-IC: The Tokyo Metropolitan Institute of Gerontology Index of Competence
